# Supplementary material for: Hot spot prediction in protein-protein interactions by an ensemble system
Source: BMC Syst Biol. 2018 Dec 31;12(Suppl 9):132. doi: 10.1186/s12918-018-0665-8 (PMC6311905; doi:10.1186/s12918-018-0665-8)
Supplement: Supplementary file 2 — All relevant MATLAB code. A simple Matlab implement of our predictor. (ZIP 19,742 kb) [file 12918_2018_665_MOESM2_ESM.zip › Additional file 2/Read ASA/ASA Property/SKEMPI/1CHOI.txt]

 NetSurfP - Protein Surface Accessibility and Secondary Structure Predictions

  

|  |  |  |
| --- | --- | --- |
|  |  | NetSurfP - Protein Surface Accessibility and Secondary Structure Predictions  Technical University of Denmark |

  


---

```
# For publication of results, please cite:
# A generic method for assignment of reliability scores applied to solvent accessibility predictions.
# Bent Petersen, Thomas Nordahl Petersen, Pernille Andersen, Morten Nielsen and Claus Lundegaard
# BMC Structural Biology 2009, 9:51 doi:10.1186/1472-6807-9-51
#
# Column 1: Class assignment - B for buried or E for Exposed - Threshold: 25% exposure, but not based on RSA
# Column 2: Amino acid
# Column 3: Sequence name
# Column 4: Amino acid number
# Column 5: Relative Surface Accessibility - RSA
# Column 6: Absolute Surface Accessibility
# Column 7: Z-fit score for RSA prediction
# Column 8: Probability for Alpha-Helix
# Column 9: Probability for Beta-strand
# Column 10: Probability for Coil
E V  1CHOI                  1    0.676 103.963  -1.414   0.005   0.015   0.979
E S  1CHOI                  2    0.621  72.840   0.442   0.005   0.262   0.733
E V  1CHOI                  3    0.294  45.142  -1.249   0.005   0.336   0.660
E D  1CHOI                  4    0.581  83.780   0.092   0.021   0.279   0.699
B C  1CHOI                  5    0.097  13.619  -1.274   0.018   0.088   0.893
E S  1CHOI                  6    0.712  83.411   0.732   0.018   0.047   0.935
E E  1CHOI                  7    0.630 110.009   0.459   0.018   0.047   0.935
B Y  1CHOI                  8    0.243  51.908   0.151   0.053   0.043   0.903
E P  1CHOI                  9    0.527  74.753   0.075   0.018   0.047   0.935
E K  1CHOI                 10    0.603 124.099  -0.377   0.018   0.088   0.893
E P  1CHOI                 11    0.314  44.571  -0.280   0.018   0.088   0.893
E A  1CHOI                 12    0.354  39.033  -0.478   0.018   0.047   0.935
B C  1CHOI                 13    0.046   6.430  -0.037   0.018   0.019   0.964
E T  1CHOI                 14    0.347  48.115  -0.256   0.053   0.043   0.903
E L  1CHOI                 15    0.425  77.799  -0.528   0.052   0.084   0.864
E E  1CHOI                 16    0.439  76.746  -0.462   0.052   0.084   0.864
B Y  1CHOI                 17    0.203  43.467   0.094   0.056   0.142   0.802
E R  1CHOI                 18    0.415  95.081  -0.357   0.052   0.084   0.864
B P  1CHOI                 19    0.115  16.375  -0.968   0.052   0.084   0.864
B L  1CHOI                 20    0.104  19.042   0.118   0.125   0.227   0.648
B C  1CHOI                 21    0.059   8.326   0.038   0.064   0.216   0.721
B G  1CHOI                 22    0.187  14.693  -1.561   0.052   0.084   0.864
E S  1CHOI                 23    0.428  50.185  -1.045   0.053   0.043   0.903
E D  1CHOI                 24    0.503  72.468  -0.597   0.018   0.019   0.964
E N  1CHOI                 25    0.381  55.837  -1.600   0.018   0.019   0.964
E K  1CHOI                 26    0.395  81.354   0.389   0.018   0.088   0.893
B T  1CHOI                 27    0.289  40.112  -0.508   0.066   0.296   0.638
B Y  1CHOI                 28    0.162  34.598   0.712   0.056   0.142   0.802
E G  1CHOI                 29    0.507  39.917  -1.030   0.184   0.043   0.773
B N  1CHOI                 30    0.245  35.897  -1.130   0.339   0.016   0.645
E K  1CHOI                 31    0.411  84.522   0.793   0.701   0.107   0.192
B C  1CHOI                 32    0.048   6.767   0.525   0.649   0.163   0.188
E N  1CHOI                 33    0.303  44.286   0.816   0.746   0.197   0.057
B F  1CHOI                 34    0.103  20.652   0.658   0.746   0.197   0.057
B C  1CHOI                 35    0.101  14.251  -0.563   0.725   0.163   0.112
B N  1CHOI                 36    0.265  38.811  -0.082   0.807   0.137   0.056
E A  1CHOI                 37    0.316  34.834  -0.361   0.725   0.163   0.112
B V  1CHOI                 38    0.289  44.358   0.581   0.680   0.265   0.055
B V  1CHOI                 39    0.162  24.823   0.494   0.578   0.229   0.194
E E  1CHOI                 40    0.574 100.348  -0.048   0.453   0.248   0.299
E S  1CHOI                 41    0.568  66.616  -0.900   0.113   0.087   0.800
E N  1CHOI                 42    0.677  99.171  -0.283   0.018   0.019   0.964
E G  1CHOI                 43    0.407  32.039  -0.423   0.018   0.047   0.935
E T  1CHOI                 44    0.505  70.016   1.267   0.005   0.336   0.660
B L  1CHOI                 45    0.043   7.873   0.246   0.003   0.718   0.279
E T  1CHOI                 46    0.345  47.838   0.964   0.002   0.816   0.182
B L  1CHOI                 47    0.115  21.111   0.837   0.001   0.900   0.099
E S  1CHOI                 48    0.385  45.157   1.065   0.001   0.900   0.099
E H  1CHOI                 49    0.303  55.098  -0.041   0.002   0.816   0.182
E F  1CHOI                 50    0.522 104.725  -0.024   0.004   0.420   0.576
E G  1CHOI                 51    0.300  23.571  -2.528   0.005   0.262   0.733
E K  1CHOI                 52    0.675 138.806   1.302   0.005   0.262   0.733
E C  1CHOI                 53    0.523  73.415  -1.924   0.003   0.003   0.994

Explain the output.  Go back.
```

---
